# The Goldilocks paradox: when partial oncoprotein inhibition fuels metastasis in Ewing sarcoma

**DOI:** 10.1038/s44321-025-00365-6

**Published:** 2026-01-03

**Authors:** Marie Castets, Adrien Bertrand-Chapel, Jean-Yves Blay

**Affiliations:** C3 Team, Consortium South-ROCK, LabEx DEVweCAN, Centre Léon Bérard, 69008 Lyon, France

**Keywords:** Cancer, Musculoskeletal System

## Abstract

JY Blay & colleagues discuss the study by Suresh et al, in this issue of *EMBO Mol Med*, that shows that incomplete inhibition of EWS::FLI1 in Ewing sarcoma paradoxically promotes metastatic behavior.

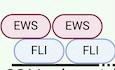

## Teaser

Targeting oncogenic fusion proteins remains a major challenge in pediatric oncology. In this issue of *EMBO Molecular Medicine*, Suresh et al (2025) demonstrate that incomplete inhibition of EWS::FLI1 in Ewing sarcoma paradoxically promotes metastatic behavior, revealing critical implications for therapeutic strategies.

Ewing sarcoma (EwS) is a high-grade sarcoma of the bone and soft tissues affecting children and young adults, driven in 85% of cases by the EWS::FLI1 (EF) fusion oncoprotein (Zöllner et al, 2021). Despite multimodal therapy, most patients with metastatic or relapsed disease develop resistance and die. The fusion protein acts as an aberrant transcription factor, rewiring the epigenome through binding to GGAA microsatellites while repressing mesenchymal differentiation programs (Riggi et al, 2010). Previous studies identified rare tumor subpopulations with low EF expression displaying enhanced migratory properties (Franzetti et al, 2017). However, the precise relationship between EF dosage and phenotypic plasticity had remained poorly understood.

**Figure 1:**
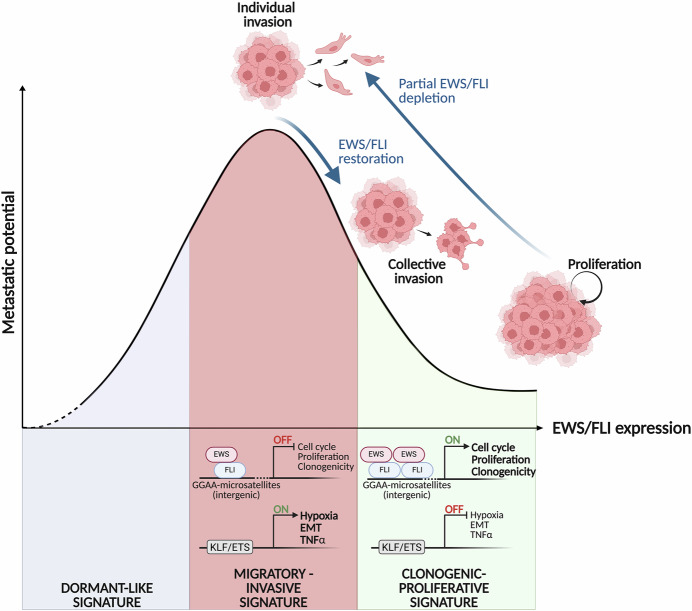
The Goldilocks model of EWS/FLI dosage in Ewing sarcoma. Metastatic potential peaks at intermediate EF levels. High EF drives clonogenic growth via GGAA microsatellite-dependent gene activation while repressing EMT programs through KLF/ETS-bound promoters. Partial EF suppression de-represses EMT, hypoxia, and TNFα signaling, maximizing individual cell invasion. Near-complete EF loss yields a dormant-like, non-tumorigenic state. EF restoration after prolonged suppression induces hybrid epithelial-mesenchymal states with collective invasion, revealing a “state memory” with therapeutic implications. Created in BioRender. Castets, M. (2025) https://BioRender.com/72r6bvj.

Using an elegant dTAG degron system enabling precise, tunable, and reversible modulation of endogenous EF levels, Suresh and colleagues establish a comprehensive dose-response framework linking oncoprotein dosage to distinct phenotypic states (Fig. 1). Their findings reveal a remarkable “Goldilocks” phenomenon, extending a concept previously proposed for EF-dependent cell growth (Seong et al, 2021): while gradual EF depletion progressively abolished clonogenic capacities, intermediate EF levels maximally enhanced migration and invasion. Intriguingly, near-complete EF loss paradoxically diminished these pro-metastatic properties, indicating that a specific dosage window drives the invasive phenotype.

Transcriptomic profiling revealed that graded EF depletion activates EMT, hypoxia, and TNF-alpha signaling programs while progressively downregulating E2F targets and cell cycle genes. Nascent RNA sequencing identified five distinct gene clusters with heterogeneous sensitivities to EF modulation. GGAA microsatellite-driven genes proved exquisitely sensitive to subtle EF reduction, consistent with the cooperative binding mechanism requiring EF multimerization at these sites (Chong et al, 2018). EF-repressed genes showed distinct derepression kinetics depending on the architecture of nearby EF-bound regions: genes near peaks enriched in canonical ETS motifs were rapidly derepressed upon EF loss, while those near promoter-associated peaks containing Krüppel-like zinc finger motifs showed a more gradual response.

Perhaps most concerning therapeutically, the authors demonstrate that transient EF fluctuations—mimicking incomplete drug-mediated inhibition—can induce persistent changes depending on the duration of EF suppression. While short-term (7-day) EF depletion was phenotypically fully reversible upon oncoprotein restoration, prolonged treatment (21 days or more) led to sustained upregulation of EMT-associated genes and retention of enhanced migratory and invasive properties that persisted even after EF recovery. In zebrafish and mouse models, transiently EF-depleted cells exhibited increased extravasation and lung metastatic burden, effects that were prevented only when EF was restored *before* injection. These observations reveal a duration-dependent “state memory” whereby prolonged exposure to intermediate EF states imprints a pro-metastatic transcriptional program.

These findings carry profound implications for EwS therapy. Multiple compounds targeting EF activity are under development, including inhibitors of EF-DNA binding and protein degraders. The present study cautions that suboptimal or transient inhibition may inadvertently promote metastatic dissemination, particularly with metronomic low-dose regimens currently considered for maintenance therapy. Collectively, Suresh et al then provide compelling evidence that therapeutic strategies must aim for complete EF eradication rather than partial inhibition. Importantly, their comprehensive characterization of EF dosage-dependent transcriptional states offers a roadmap for identifying specific vulnerabilities associated with each phenotypic state. This opens the possibility for rationally designed combination therapies that could target cells transitioning through pro-metastatic intermediates, potentially preventing escape into treatment-resistant, dissemination-prone states.
